# The CSF neurofilament light signature in rapidly progressive neurodegenerative dementias

**DOI:** 10.1186/s13195-017-0331-1

**Published:** 2018-01-11

**Authors:** Samir Abu-Rumeileh, Sabina Capellari, Michelangelo Stanzani-Maserati, Barbara Polischi, Paolo Martinelli, Paola Caroppo, Anna Ladogana, Piero Parchi

**Affiliations:** 1 Department of Biomedical and NeuroMotor Sciences, University of Bologna, 40123 Bologna, Italy; 2 Istituto di Ricovero e Cura a Carattere Scientifico (IRCCS) Institute of Neurological Sciences of Bologna, Bellaria Hospital, 40139 Bologna, Italy; 3 Istituto di Ricovero e Cura a Carattere Scientifico (IRCCS) Foundation “Carlo Besta” Neurological Institute, 20133 Milan, Italy; 4 Department of Neurosciences, Istituto Superiore di Sanità, Rome, Italy; 5 Department of Diagnostic Experimental and Specialty Medicine (DIMES), University of Bologna, 40138 Bologna, Italy

**Keywords:** Frontotemporal dementia, Progressive supranuclear palsy, Corticobasal syndrome, Creutzfeldt-Jakob disease, Alzheimer’s disease, Dementia with Lewy bodies

## Abstract

**Background:**

Neurofilament light chain protein (NfL) is a surrogate biomarker of neurodegeneration that has never been systematically tested, either alone or in combination with other biomarkers, in atypical/rapidly progressive neurodegenerative dementias (NDs).

**Methods:**

Using validated, commercially available enzyme-linked immunosorbent assay kits, we measured cerebrospinal fluid (CSF) NfL, total tau (t-tau), phosphorylated tau, and β-amyloid 42 in subjects with a neuropathological or clinical diagnosis of prion disease (*n* = 141), Alzheimer’s disease (AD) (*n* = 73), dementia with Lewy bodies (DLB) (*n* = 35), or frontotemporal lobar degeneration (FTLD) (*n* = 44). Several cases with an atypical/rapidly progressive course were included in each group. We evaluated the diagnostic accuracy of every CSF biomarker and their combinations by ROC curve analyses.

**Results:**

In each patient group CSF NfL showed higher levels than in control subjects, reaching the highest values in those with Creutzfeldt-Jakob disease (CJD). In the latter, NfL showed a divergent, subtype-specific correlation with t-tau, depending on the degree of subcortical involvement and disease duration. Most significantly, patients with classic sporadic CJD (sCJD) MM1 showed a significantly lower concentration of CSF NfL than those with sCJD MV2, despite the much higher t-tau levels and the more rapid clinical course. High NfL levels were also detected in most atypical CJD cases, showing a disease duration longer than 2 years and/or borderline/negative results in other CSF assays (e.g., 14-3-3, t-tau, and prion real-time quaking-induced conversion). Rapidly progressive/atypical cases showed higher NfL levels than typical patients in FTLD, but not in AD or DLB. NfL showed accuracy similar to that of t-tau in discriminating CJD from other NDs, but it had higher efficacy in differentiating atypical forms, especially in regard to Alzheimer’s disease.

**Conclusions:**

The present data indicate that CSF NfL and t-tau levels reflect distinct pathophysiological mechanisms of neurodegeneration and support the clinical use of NfL as a fast screening biomarker for the differential diagnosis of atypical/rapidly progressive NDs.

**Electronic supplementary material:**

The online version of this article (doi:10.1186/s13195-017-0331-1) contains supplementary material, which is available to authorized users.

## Background

Prion diseases are rapidly progressive and highly heterogeneous neurodegenerative disorders encompassing four major phenotypic entities, namely, Creutzfeldt-Jakob disease (CJD), Gerstmann-Sträussler-Scheinker syndrome (GSS), fatal familial insomnia, and variably protease-sensitive prionopathy (VPSPr) [1, 2]. CJD, by far the most common form, includes six major clinicopathological subtypes that are determined largely by the genotype at the methionine (M)/valine (V) polymorphic codon 129 of the *PRNP* gene and the type (1 or 2) of disease-associated prion protein (PrP^Sc^) accumulating in the brain, namely MM(V)1, MM2 cortical (MM2C), MM2 thalamic (MM2T), MV2 kuru type (MV2K), VV1, and VV2 [3].

Alzheimer’s disease (AD), frontotemporal lobar degeneration (FTLD), and dementia with Lewy bodies (DLB) are neurodegenerative diseases that typically show a slowly progressive cognitive and/or motor decline. However, on one hand, variants of these neurodegenerative dementias (NDs) may sometimes present with an atypical rapid course and may even show periodic sharp wave complexes (PSWCs) on electroencephalographic (EEG) examination [4–8]. On the other hand, owing to the wide phenotypic heterogeneity, a clinical presentation mimicking AD, DLB, frontotemporal dementia (FTD), corticobasal syndrome (CBS), or progressive supranuclear palsy (PSP) may occasionally be sustained by a prion disease, especially by the least rapidly evolving variants (e.g., CJD MV2, MM2, VV1, VPSPr, or GSS) [1, 2, 4, 8–11]. Established biomarkers for the clinical in vivo diagnosis of prion disease are cerebrospinal fluid (CSF) 14-3-3 protein; total (t)-tau; t-tau/phosphorylated (p)-tau (or p-tau/t-tau) ratio; and, more recently, the prion real-time quaking-induced conversion (RT-QuIC) assay [8, 12–14]. However, given the wide heterogeneity of prion disease, which is reflected in variable CSF protein levels among the different disease subtypes, the diagnostic sensitivity of these biomarkers is still not optimal [8, 13–15]. Furthermore, NDs may sometimes present CSF protein values significantly overlapping with those detected in prion disease regarding CSF t-tau and 14-3-3 [7, 8, 13, 14, 16, 17]. In this respect, researchers in several studies have analyzed the diagnostic value of the combined analyses of multiple CSF protein markers, including t-tau, p-tau, β-amyloid 42 (Aβ_42_), and total prion protein, and these studies revealed improved performance in terms of sensitivity and specificity [8, 13, 14, 17, 18].

Neurofilament light chain proteins (NfLs) are located mainly in the axoplasm of large myelinated neurons and play an important role in maintaining neuronal structure [19, 20]. CSF NfL levels represent a surrogate biomarker of neuroaxonal degeneration [19]. To date, CSF and/or blood NfL in prion diseases have been investigated in only two studies [21, 22]. Although both studies disclosed higher NfL levels in CJD than in other NDs, these results need to be validated in a wider patient population, including all prion disease variants as well as atypical cases. Similar analyses were also conducted in populations of patients with AD, DLB, and FTLD [22–29]. However, the diagnostic role of NfL in a clinically and biologically based cohort of rapidly progressive or atypical (either clinically or for the CSF profile mimicking CJD) NDs has not been investigated yet.

In the present study, we systematically analyzed CSF NfL levels in different prion diseases according to molecular subtypes, in AD, DLB, and FTLD. Furthermore, we explored the value of NfL alone or in several combinations with t-tau, p-tau, and Aβ_42_ in the differential diagnosis of NDs, focusing on rapidly progressive/atypical variants.

## Methods

### Study cohort

We retrospectively analyzed 323 CSF samples submitted for analysis of rapidly progressive/atypical dementias to the Neuropathology Laboratory at the Institute of Neurological Sciences of Bologna (*n* = 300) or to other national reference laboratories from 2009 to 2016. Specifically, the cohort comprised 30 cognitively healthy control subjects and 293 patients with a diagnosis of ND, including 141 with prion disease, 73 with AD, 35 with DLB, and 44 with FTLD.

The study was conducted according to the revised Declaration of Helsinki and good clinical practice guidelines. Informed consent was provided by study participants or their next of kin. Data collection for clinically suspected cases is an integral part of the national CJD surveillance study, which was approved by the ethics committee of the Istituto Superiore di Sanità (CE-ISS 09/266; 29 May 2009).

For each patient, we collected and reviewed data regarding clinical history and neurological examinations, including the evaluation of cognitive status. Moreover, we obtained results of Mini Mental State Examination and/or specific neuropsychological test batteries for the large majority of testable patients. We also retrieved the results of EEG recordings, brain computed tomography (CT), and brain magnetic resonance imaging (MRI), inclusive of fluid-attenuated inversion recovery and diffusion-weighted (DW) imaging sequences, and/or fluorodeoxyglucose-positron emission tomography and/or cerebral blood flow single-photon emission computed tomography.

Patients with prion diseases were classified into diagnostic categories according to the most recently updated World Health Organization diagnostic criteria for the diagnosis of CJD and related disorders [12]. Briefly, patients with “definite” prion disease consisted of 98 autopsy-confirmed prion cases (97 sporadic Creutzfeldt-Jakob disease [sCJD], 1 VPSPr) and genetic cases carrying a pathogenic *PRNP* mutation (16 genetic Creutzfeldt-Jakob disease [gCJD], 1 GSS), whereas the group with “probable” prion diseases included 27 patients fulfilling the clinical criteria for possible CJD and showing either a positive EEG study or a positive DW-MRI scan result.

We carried out a molecular analysis of the *PRNP* gene in all subjects with a definite or probable diagnosis of prion disease, as previously described [3]. Moreover, PrP^Sc^ typing and CJD histotype classification were performed for all autopsied cases according to established methodologies and consensus criteria [30, 31].

Furthermore, in all cases with a positive familial history for dementia and those with a clinical history compatible with early-onset ND (aged < 60 years; *n* = 69), we screened for variants in 22 dementia-associated genes using the MiSeq sequencer with the TruSeq Custom Amplicon version 1.5 amplicon-based assay (Illumina, San Diego, CA, USA), as described by Beck et al. [32]. Major screened genes included *PSEN1*, *PSEN2*, *APP*, *PRNP*, *GRN*, *MAPT*, *TARDBP*, and *FUS*. In addition, in the same patient group, we screened for the presence of the *C9orf72* repeat expansion using a two-step strategy with Southern blotting confirmation, as previously described [33].

The clinical diagnosis of AD was made according to the 2011 National Institute on Aging-Alzheimer’s Association workgroup guidelines [34]. In particular, after a clinical follow-up of at least 24 months, all patients with AD (*n* = 73) fulfilled the criteria for probable AD dementia with high evidence of the AD pathophysiological process. Moreover, in the five autopsied cases, neuropathological assessment revealed an intermediate or high degree of AD pathology [35], whereas in one clinical case, genetic screening showed a pathogenic mutation in *PSEN1*.

The clinical diagnosis of DLB, FTD (behavioral variant of frontotemporal dementia [bvFTD] and primary progressive aphasia [PPA]), CBS, and PSP was also made according to established criteria [36–40]. The cohort included 35 DLB cases, 11 of which had a neuropathological diagnosis; 25 FTD cases (19 bvFTD and 6 PPA); 11 CBS cases; and 8 PSP cases. The clinical diagnosis was confirmed neuropathologically in one PSP case. The clinical diagnosis was strongly supported by the finding of a pathogenic mutation in *GRN* (*n* = 4), *FUS* (*n* = 2), *MAPT* (*n* = 2), *TARDBP* (*n* = 1), and *C9orf72* (*n* = 1) in 10 FTLD cases. Finally, in all remaining FTLD cases, the AD pathophysiological process was excluded on the basis of findings of normal levels of CSF p-tau and Aβ_42_.

In both ND and prion disease groups, we selected a significant number of patients with an atypical clinical presentation and/or an atypical CSF biomarker profile. Three consultant neurologists (SAR, SC, and PP) determined the clinical diagnosis and the classification as “typical” or “atypical/rapidly progressive” by majority consensus while blinded to the results of CSF NfL measurement after reviewing clinical features, CSF biomarker values, and the results of EEG and neuroimaging investigations. Specifically, we defined as atypical prion disease each case presenting at least one of the following features: (1) clinical course > 2 years, (2) progressive cognitive decline without focal neurological signs (up to the time of CSF analysis), (3) CSF t-tau < 1100 pg/ml, and/or (4) borderline or negative CSF 14-3-3 assay.

Similarly, the classification of atypical/rapidly progressive ND (all diagnostic groups) required at least one of the following: (1) rapid cognitive decline leading to the clinical suspicion of prion disease, (2) CSF t-tau > 1100 pg/ml, (3) a positive CSF 14-3-3 assay or (4) presence of PSWCs at EEG evaluation. Furthermore, for AD cases only, the presence of motor signs at the time of CSF analysis was also considered a further criterion [8, 17].

The control group included 30 age- and sex-matched subjects lacking any clinical or neuroradiological evidence of central nervous system disease (i.e., minor psychiatric disorders, noninflammatory peripheral neuropathies, tension-type headache). In the cohort there were 59 patients with prion disease, 37 with AD, 11 with DLB, and 9 with FTLD manifesting an atypical and/or rapidly progressive clinical course and/or showing an atypical CSF biomarker profile (Table 1). All the remaining patients were classified as “typical.”

**Table 1:** Clinical, laboratory and electroencephalographic features of cases classified as atypical/rapidly progressive neurodegenerative dementia

	a/rpAD	a/rpDLB	a/rpFTLD
No. of subjects	37	11	9
Clinical presentation			
Cognitive decline	37/37	11/11	9/9
Extrapyramidal signs	5/37	11/11	3/9
Pyramidal signs	3/37	0/11	1/9
Myoclonus	5/37	3/11	3/9
Akinetic mutism	5/37	1/11	2/9
Biomarker data			
t-tau > 1100 pg/ml	19/37	5/10	2/9
Positive 14-3-3	4/37	3/10	1/9
EEG PSWC	5/37	4/11	1/9

*Abbreviations: AD* Alzheimer’s disease, *a/rp* Atypical/rapidly progressive, *DLB* Dementia with Lewy bodies, *EEG* Electroencephalographic, *FTLD* Frontotemporal lobar degeneration, *PSWC* Periodic sharp wave complexes, *t-tau* Total tau protein

### CSF biochemical analysis

CSF samples were obtained by lumbar puncture (LP) at the L3/L4 or L4/L5 level following a standard procedure, divided into aliquots, and stored in polypropylene tubes at −80 °C until analysis. CSF levels of proteins 14-3-3, t-tau, p-tau, Aβ_42_, and NfL were analyzed in all cases. The 14-3-3 protein was detected by Western blotting using CSF control subjects with a weak or strong 14-3-3 signal as internal quality control subjects, as described previously [14].

CSF t-tau, p-tau, and Aβ_42_ levels were analyzed using commercially available enzyme-linked immunosorbent assay (ELISA) kits (INNOTEST htau-Ag, INNOTEST phosphorylated-Tau181, and INNOTEST Aβ1–42; Innogenetics/Fujirebio Europe, Ghent, Belgium) according to the manufacturer’s instructions. The optimal cutoff value for t-tau was chosen after analyzing the distribution of sensitivity and specificity at different decision points and calculated as 1100 pg/ml on the basis of maximum potential effectiveness (Youden index 0.73). PrP^Sc^ seeding activity was detected by RT-QuIC as previously described [14].

NfL protein levels were analyzed using a commercially available ELISA kit (IBL, Hamburg, Germany) according to the manufacturer’s specifications. The measurement of NfL in samples from the same patients (*n* = 5) with repeated (up to three) freeze-thaw cycles did not show a significant reduction in protein levels, in agreement with previous studies showing that Nf proteins are stable under the most prevalent preanalytical variations [41]. The mean interassay coefficient of variation for the ELISAs (all assays) was < 20%.

### Statistical analyses

Statistical analysis was performed using IBM SPSS Statistics version 21 software (IBM, Armonk, NY, USA). Several combinations of biomarkers were analyzed. Depending on the data distribution, the Mann-Whitney *U* test or the *t* test was used to test differences between two groups, whereas the Kruskal-Wallis test or one-way analysis of variance (followed by Tukey’s post hoc test) was applied for multiple group comparisons. Data were expressed as mean ± SD or median and IQR on the basis of analysis of the distribution of values (normal or nonnormal distribution, respectively). The Bonferroni correction was applied to analysis with multiple comparisons. ROC curve analyses were performed to establish the diagnostic accuracy, sensitivity, and specificity of each biomarker or combination of biomarkers. The optimal cutoff value for biomarkers was chosen using the maximized Youden index. The Youden index for a cutoff is defined by its sensitivity + specificity − 1. The Spearman bivariate test was used to detect the strength of correlation between some of the analyzed variables. Differences were considered statistically significant at *p* < 0.05.

## Results

Demographic data and classification of patient groups are shown in Table 2.

**Table 2:** Demographics and classification of patient groups

	Total (*n*)	Typical cases (*n*)	Atypical cases (*n*)	Age at LP^a^, years (mean ± SD)	Female sex (%)
Prion diseases	141	82	59	65.5 ± 9.9	55.6
Definite sCJD	97	62	35		
MM(V)1	37	30	7		
VV2	26	26	0		
MV2K	22	6	16		
MM2C	8	0	8		
MM2T	2	0	2		
VV1	1	0	1		
VPSPr (VV)	1	0	1		
Definite gCJD	16	7	9		
E200K-129 M	11	4	7		
V210I-129 M	4	3	1		
D178N-129 V	1	0	1		
Probable CJD	27	13	14		
MM	4	1	3		
MV	14	3	11		
VV	9	9	0		
GSS	1	0	1		
AD	73	36	37	66.9 ± 9.5	61.6
DLB	35	24	11	72.3 ± 7.8	40.0
FTLD	44	35	9	63.0 ± 9.0	43.2
FTD	25	19	6		
bvFTD	19	14	5		
PPA	6	5	1		
CBS	11	9	2		
PSP	8	7	1		
Control subjects	30			63.6 ± 10.7	36.7

*Abbreviations: AD* Alzheimer’s disease, *bvFTD* Behavioral variant of frontotemporal dementia, *CBS* Corticobasal syndrome, *CJD* Creutzfeldt-Jakob disease, *DLB* Dementia with Lewy bodies, *FTLD* Frontotemporal lobar degeneration, *gCJD* Genetic Creutzfeldt-Jakob disease, *GSS* Gerstmann-Sträussler-Scheinker syndrome, *LP* Lumbar puncture, *MM(V)1* Methionine homozygosity (valine) and scrapie prion protein type 1, *MM2C* Methionine homozygosity and scrapie prion protein type 2, cortical type, *MM2T* Methionine homozygosity and scrapie prion protein type 2, thalamic type, *MV2K* Methionine/valine heterozygosity and scrapie prion protein type 2, kuru type, *PPA* Primary progressive aphasia, *PSP* Progressive supranuclear palsy, *sCJD* Sporadic Creutzfeldt-Jakob disease, *VPSPr* Variably protease-sensitive prionopathy, *VV1* Valine homozygosity and scrapie prion protein type 1, *VV2* Valine homozygosity and scrapie prion protein type 2

^a^No significant differences regarding age were detected between groups by one-way analysis of variance (followed by Tukey’s post hoc test) with the Bonferroni correction

Owing to nonnormal distribution of biomarker values and the presence of outliers, Mann-Whitney *U* and Kruskal-Wallis tests (followed by the Bonferroni correction) were performed for multiple comparisons between two or more patient groups.

### CSF NfL, t-tau, p-tau, and Aβ_42_ levels in the diagnostic groups

The results of biomarker analyses according to diagnostic groups are summarized in Table 3.

**Table 3:** Cerebrospinal fluid biomarker data in all groups

		NfL (pg/ml) Median (IQR)	t-tau^a^ (pg/ml) Median (IQR)	p-tau^b^ (pg/ml) Median (IQR)	Aβ_42_^c^ (pg/ml) Median (IQR)	t-tau/p-tau Median (IQR)	NfL/p-tau Median (IQR)	14-3-3 (positive)
Prion disease	Typical (*n* = 82)	15,000 (9254–24,425)	7048 (3549–10,550)	60 (47–75)	646 (420–811)	121.60 (66.18–194.90)	257.89 (177.94–402.00)	82/82
	Atypical (*n* = 59)	9139 (5234–17,000)	1546 (933–2221)	44 (34–63)	527 (372–771)	33.57 (21.43–54.91)	215.98 (125.26–425.00)	21/59
	Total prion disease (*n* = 141)	12,300 (7160–22,650)	3103 (1803–8555)	51 (40–71)	620 (399–780)	66.94 (33.57–152.67)	247.29 (156.02–404.71)	103/141
AD	Typical (*n* = 36)	1933 (1515–2788)	572 (424–817)	80 (67–105)	363 (277–437)	7.09 (6.11–7.80)	23.55 (18.48–30.27)	0/36
	Atypical (*n* = 37)	2521 (1662–3330)	1115 (772–1631)	123 (102–153)	352 (257–508)	8.90 (7.85–10.32)	17.39 (12.86–28.85)	4/37
	Total AD (*n* = 73)	2033 (1592–3067)	822 (565–1186)	106 (74–143)	358 (268–465)	7.84 (6.71–9.72)	20.30 (14.85–29.77)	4/73
DLB	Typical (*n* = 24)	1857 (1398–2682)	268 (125–395)	42 (34–54)	647 (334–912)	5.63 (4.76–7.18)	41.85 (30.39–61.69)	0/22
	Atypical (*n* = 11)	4207 (1633–29,500)	713 (230–1374)	51 (31–112)	360 (179–559)	7.43 (5.10–19.91)	88.91 (19.10–573.97)	3/10
	Total DLB (*n* = 35)	2171 (1414–4007)	275 (160–438)	42 (33–61)	476 (303–722)	6.20 (4.89–8.18)	42.34 (28.68–94.20)	3/32
FTLD	Typical (*n* = 35)	3191 (1910–4963)	217 (154–378)	38 (27–53)	711 (570–928)	6.12 (4.40–7.91)	78.00 (40.13–158.08)	0/31
	Atypical (*n* = 9)	6785 (3785–12,500)	341 (198–849)	45 (31–52)	708 (372–877)	7.80 (6.02–10.42)	153.54 (72.90–364.83)	1/9
	Total FTLD (*n* = 44)	3628 (2308–6647)	245 (173–407)	41 (28–53)	709 (546–920)	6.52 (4.76–9.02)	86.06 (43.11–197.90)	1/40
	bvFTD (*n* = 19)	3729 (1910–11,900)	204 (150–380)	35 (26–44)	717 (519–922)	6.52 (4.69–7.80)	153.54 (41.24–304.65)	1/17
	PPA (*n* = 6)	5626 (2768–8173)	393 (177–637)	47 (34–62)	928 (744–1214)	7.78 (5.83–10.71)	109.11 (63.12–177.75)	0/6
	Tauopathies (CBS + PSP) (*n* = 19)	2733 (2459–3940)	244 (177–373)	45 (28–57)	619 (452–785)	6.54 (4.30–8.13)	60.73 (40.13–138.74)	0/17
Control subjects	Total (*n* = 30)	1167 (805–1543)	164 (136–255)	38 (30–46)	815 (642–1045)	5.43 (4.06–6.20)	33.00 2(20.22–44.26)	NA

*Abbreviations: Aβ42* β-Amyloid 42, *AD* Alzheimer’s disease, *bvFTD* Behavioral variant of frontotemporal dementia, *CBS* Corticobasal syndrome; *DLB* Dementia with Lewy bodies, *FTLD* Frontotemporal lobar degeneration, *NA* Not available, *NfL* Neurofilament light chain protein, *PPA* Primary progressive aphasia, *PSP* Progressive supranuclear palsy, *p-tau* Phosphorylated tau protein, *t-tau* Total tau protein

^a^Prion disease vs. control subjects (*p* < 0.001); prion disease vs. each ND group (*p* < 0.001); AD vs. control subjects (*p* < 0.001); AD vs. each ND group (*p* < 0.001)

^**b**^AD vs. prion disease (*p* < 0.001); prion disease vs. FTLD (*p* < 0.001); prion disease vs. bvFTD (*p* < 0.001); AD vs. FTLD (*p* < 0.001); AD vs. bvFTD or PPA or tauopathies (*p* < 0.001)

^c^Prion disease vs. AD (*p* < 0.001); FTLD vs. DLB (*p* = 0.018); DLB vs. PPA (*p* = 0.004); AD vs. FTLD (*p* < 0.001); AD vs. bvFTD or PPA or tauopathies (*p* < 0.001); AD vs. DLB (*p* = 0.024)

All patients with NDs showed significantly increased CSF NfL levels compared with control subjects (*p* < 0.001 for each ND group vs. control subjects) (Table 3). CSF NfL levels were significantly higher in patients with prion disease than in patients with AD, DLB, or FTLD (*p* < 0.001 for all comparisons). There were also statistical differences regarding CSF NfL levels between FTLD and AD (*p* < 0.001) or DLB (*p* = 0.019), with a lower protein concentration in AD or DLB than in FTLD. In detail, NfL levels were significantly different between AD and bvFTD (*p* = 0.004), AD and PPA (*p* = 0.012), AD and tauopathies (*p* = 0.01), and DLB and bvFTD (*p* = 0.04). No other comparisons were statistically significant.

CSF t-tau levels significantly differed between prion disease and AD cases and between both prion disease and AD and all other ND groups (Table 3). Statistically significant differences in p-tau levels were detected between prion disease and AD, between prion disease and FTLD, and between AD and all other ND groups (*p* < 0.001 in all comparisons) (Table 3). Aβ_42_ levels were significantly different between prion disease and AD, between FTLD and DLB, and between AD and all other ND groups (Table 3). Finally, recognizing the limits of the analysis, given the small sample sizes, NfL, t-tau, p-tau, and Aβ_42_ levels did not significantly differ between bvFTD, PPA, and tauopathies (CBS + PSP).

### CSF biomarker values in prion diseases

The demographic characteristics and results of CSF biomarkers for the different subtypes of prion disease are summarized in Table 4.

**Table 4:** Histotype classification, demographic features, and biomarker values of prion disease cases

	No. of subjects	Time from onset to LP (months ± SD)	Disease duration (months ± SD)	NfL, pg/ml, median (IQR)	t-tau^a^, pg/ml, median (IQR)	p-tau, pg/ml, median (IQR)	14-3-3 (positive)	RT-QuIC (positive)
Definite sCJD	97							
MM(V)1	37	2.0 ± 2.0	3.6 ± 2.8	9600 (6507–14,750)	6388 (2255–9258)	47 (37–59)	33/37	32/37
VV2	26	3.8 ± 1.2	5.5 ± 2.2	22,800 (14,050–30,550)	9729 (5334–14,850)	71 (62–95)	25/26	22/26
MV2K	22	7.3 ± 4.2	18.8 ± 13.5	16,100 (9650–24,275)	1972 (1454–2789)	55 (43–88)	12/22	18/22
MM2C	8	10.4 ± 7.1	22.8 ± 14.0	8808 (6558–9908)	1457 (821–2488)	37 (19–64)	3/8	4/8
MM2T	2	12; 13	24; 36	12,100; 7959	630; 102	20; 25	0/2	1/2
VV1	1	10	13.5	31,800	3790	49	1/1	1/1
VPSPr (VV)	1	3	36	3212	1273	140	1/1	0/1
Definite gCJD	16							
E200K-129M	11	3.2 ± 2.5	15.3 ± 15	9088 (5976–15,000)	1881 (892–2955)	34 (26–48)	7/11	11/11
V210I-129M	4	4.1 ± 4.0	7.0 ± 6.0	5587 (4438–6772)	4907 (2316–7148)	36 (30–36)	3/4	4/4
D178N-129V	1	3.5	Alive	4909	2206	44	1/1	0/1
Probable CJD	27							
MM	4	2.7 ± 2.6	–	8345 (5345–18,323)	3393 (797–4633)	44 (34–62)	2/4	3/4
MV	14	11.0 ± 8.2	–	14,700 (6163–23,425)	2042 (1260–3709)	68 (47–87)	5/14	10/14
VV	9	2.8 ± 1.0	–	27,400 (16,500–35,200)	13,300 (4544–16,350)	82 (58–103)	9/9	7/9
GSS	1	18	Alive	5221	566	NA	0/1	0/1

*Abbreviations: CJD* Creutzfeldt-Jakob disease, *gCJD* Genetic Creutzfeldt-Jakob disease, *GSS* Gerstmann-Sträussler-Scheinker syndrome, *LP* Lumbar puncture, *MM(V)1* Methionine homozygosity (valine) and scrapie prion protein type 1, *MM2C* Methionine homozygosity and scrapie prion protein type 2, cortical type, *MM2T* Methionine homozygosity and scrapie prion protein type 2, thalamic type, *MV2K* Methionine/valine heterozygosity and scrapie prion protein type 2, kuru type, *NfL* Neurofilament light chain protein, *p-tau* Phosphorylated tau protein, *RT-QuIC* Real-time quaking-induced conversion, *sCJD* Sporadic Creutzfeldt-Jakob disease, *t-tau* Total tau protein, *VPSPr* Variably protease-sensitive prionopathy, *VV1* Valine homozygosity and scrapie prion protein type 1, *VV2* Valine homozygosity and scrapie prion protein type 2

^a^t-tau: VV2 vs. MV2K or MM2C (*p* < 0.001); MM(V)1 vs. MV2K or MM2C (*p* < 0.001)

CSF NfL levels varied significantly between prion disease subtypes (Table 4). Specifically, sCJD VV2 cases demonstrated the highest NfL levels, followed by MV2K and to a larger extent by MM(V)1, MM2C, and MM2T cases (VV2 vs. MM1 *p*<0.001; VV2 vs. MV2K *p*=0.066; VV2 vs. MM2C *p*<0.001; MM(V)1 vs. MV2K *p*=0.015; MM(V)1 vs. MM2C *p*=0.272; MV2K vs. MM2C *p*=0.008). The single case of VV1 showed one of the highest NfL values (31,800 pg/ml), whereas MM2T and VPSPr cases showed the lowest levels. In gCJD, there were no significant differences between V210I-129 M and E200K-129 M carriers, but the sample sizes were very small. The GSS cases showed a lower NfL level (5221 pg/ml) than most CJD cases. NfL levels were also high in prion cases with negative 14-3-3 and/or RT-QuIC assays. As previously shown [14], VV2 and MM1 cases showed significantly higher t-tau levels than MV2K and MM2C cases (Table 4). Atypical prion disease cases showed lower levels of NfL, t-tau, and p-tau than typical ones (*p* < 0.001 for every comparison) (Table 3).

### Correlations between biomarker values and demographic variables in definite prion cases

These analyses were performed in the largest homogeneous prion disease groups, specifically definite sporadic MM(V)1, VV2, and MV2K, because it is well established that the CJD subtype has a profound effect on disease duration. In the group of definite sporadic MM(V)1, there was an effect of time from clinical onset to LP on NfL (Spearman’s rho = 0.349, *p* = 0.03). In the same group, t-tau levels correlated with NfL (Spearman’s rho = 0.618, *p* < 0.001) and p-tau levels (Spearman’s rho = 0.478, *p* = 0.003). There was no effect of time from clinical onset to LP and disease duration on other biomarker values.

In the groups of definite VV2 and MV2K, there was no effect of time from clinical onset to LP and disease duration on NfL, t-tau, p-tau, and Aβ_42_ levels. In both groups, t-tau correlated with p-tau levels (Spearman’s rho = 0.431, *p* = 0.045; Spearman’s rho = 0.669, *p* = 0.002). Moreover, only in the group of VV2 was there a slight tendency toward a positive correlation between t-tau and NfL (Spearman’s rho = 0.383, *p* = 0.06).

### CSF biomarker values in typical and atypical/rapidly progressive NDs

Patients with atypical/rapidly progressive AD showed higher levels of t-tau (*p* < 0.001) and p-tau (*p* < 0.001) than typical cases, although no differences regarding NfL levels and Aβ_42_ were detected (Table 3). No differences regarding NfL, t-tau, p-tau, and Aβ_42_ were noted between atypical/rapidly progressive and typical DLB cases. Atypical/rapidly progressive FTLD cases showed higher levels of NfL (*p* = 0.032) but no differences regarding t-tau, p-tau, and Aβ_42_ levels.

### Diagnostic accuracy of CSF biomarkers for differentiation between ND cases and control subjects

The diagnostic value in the differentiation between prion cases and control subjects was excellent for NfL (AUC 1.000 ± 0.001), t-tau (AUC 0.991 ± 0.008), tau/p-tau (AUC 0.994 ± 0.006), and NfL/p-tau (AUC 0.984 ± 0.009). In the comparison between AD or FTLD cases and control subjects, the highest accuracy was reached by t-tau (AUC 0.993 ± 0.006) and NfL (0.927 ± 0.031), respectively. Further comparisons are shown in Additional file 1: Table S1.

### Diagnostic accuracy of CSF biomarkers in the differential diagnosis of NDs

Results of the ROC analysis for biomarker combinations are shown in Table 5.

**Table 5:** Diagnostic value of cerebrospinal fluid biomarkers in differential diagnosis of prion disease

	Prion disease vs. other NDs					Atypical prion disease vs. other a/rpNDs				
	AUC		Cutoff	Sensitivity (%)	Specificity (%)	AUC		Cutoff	Sensitivity (%)	Specificity (%)
NfL	0.926 ± 0.016	>	5016 pg/ml	89.2	83.4	0.839 ± 0.040	>	4500 pg/ml	85.5	75
t-tau	0.939 ± 0.014	>	1100 pg/ml	89.2	84.1	0.722 ± 0.048	>	1100 pg/ml	74.5	57.1
t-tau/p-tau	0.982 ± 0.009	>	14.0	96.2	95.4	0.930 ± 0.028	>	14.0	89.1	89.3
NfL/p-tau	0.904 ± 0.019	>	113.0	87.7	83.4	0.866 ± 0.039	>	95.0	89.1	82.1
	Prion disease vs. AD					Atypical prion disease vs. a/rpAD				
	AUC		Cutoff	Sensitivity (%)	Specificity (%)	AUC		Cutoff	Sensitivity (%)	Specificity (%)
NfL	0.981 ± 0.007	>	4363 pg/ml	94.3	94.5	0.946 ± 0.021	>	4363 pg/ml	86.4	91.9
t-tau	0.901 ± 0.021	>	1100 pg/ml	89.1	69.9	0.654 ± 0.057	>	1100 pg/ml	75.9	48.6
t-tau/p-tau	0.986 ± 0.008	>	14.0	94.7	95.9	0.958 ± 0.022	>	14.0	89.3	94.6
NfL/p-tau	0.994 ± 0.004	>	60.0	96.2	95.9	0.989 ± 0.007	>	60.0	92.9	97.3

*Abbreviations: AD* Alzheimer’s disease, *a/rp* Atypical/rapidly progressive, *ND* Neurodegenerative dementia, *NfL* Neurofilament light chain protein, *p-tau* Phosphorylated tau protein, *t-tau* Total tau protein, > greater than

**Fig. 1:**
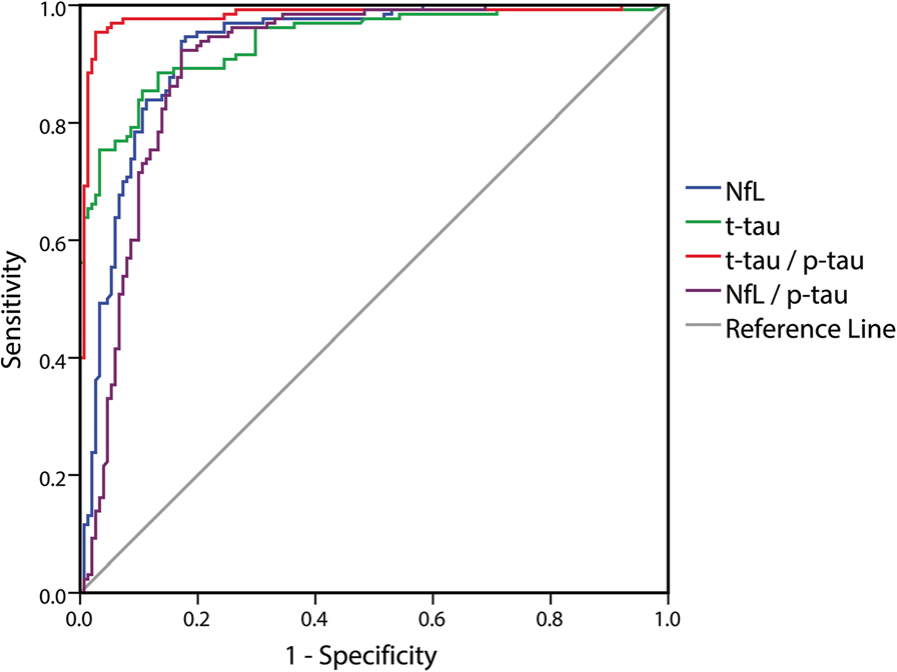
ROC analysis for cerebrospinal fluid (CSF) biomarkers in the comparison between prion disease and other neurodegenerative dementias (NDs). ROC curves illustrate sensitivity and specificity of various CSF biomarker combinations in the differential diagnosis between prion disease and other NDs. AUC values are reported. The corresponding AUC values are also listed in Table 5. *NfL* Neurofilament light chain protein, *p-tau* Phosphorylated tau protein, *t-tau* Total tau protein

The diagnostic power of NfL (AUC 0.926 ± 0.016) and t-tau (AUC 0.939 ± 0.014) was comparable in terms of sensitivity and specificity in the overall discrimination of prion disease from other NDs, whereas the t-tau/p-tau ratio showed the highest accuracy in this differential diagnosis (AUC 0.982 ± 0.09) (Fig. 1). However, in the specific comparison between prion disease and AD, NfL showed higher diagnostic value (AUC 0.981 ± 0.007) than t-tau (AUC 0.901 ± 0.021), and the NfL/p-tau ratio yielded the highest accuracy (AUC 0.994 ± 0.004), with 96.2% sensitivity and 95.9% specificity (*see* Additional file 1: Figure S1).

Otherwise, in the specific comparisons between prion disease and DLB and between prion disease and FTLD, the diagnostic accuracy of t-tau (AUC 0.963 ± 0.015; 0.982 ± 0.009) was superior to that of NfL (AUC 0.880 ± 0.047; 0.870 ± 0.032), and the t-tau/p-tau ratio (AUC 0.970 ± 0.019; 0.983 ± 0.010) was the best biomarker (*see* Additional file 1: Table S2). In the differential diagnosis between AD and other NDs (FTLD and DLB), NfL accuracy (AUC 0.623 ± 0.046) was quite lower than that of Aβ_42_, t-tau, and p-tau alone (AUC from 0.775 to 0.919) (Additional file 1: Table S2). However, the NfL × Aβ_42_/p-tau ratio best distinguished FTLD from other NDs (AD and DLB) (AUC 0.900 ± 0.025) and especially FTLD from AD (AUC 0.975 ± 0.011) (*see* Additional file 1: Table S2).

### Diagnostic accuracy of CSF biomarkers in the differential diagnosis of rapidly progressive NDs

NfL performed better than t-tau (AUC 0.839 ± 0.040 vs. 0.722 ± 0.048) in the discrimination of atypical prion disease from other atypical/rapidly progressive NDs, although overall the t-tau/p-tau ratio had the best accuracy (AUC 0.930 ± 0.028) (Table 5; *see also* Additional file 1: Figure S2). However, NfL was superior to t-tau in the discrimination between atypical prion disease and atypical/rapidly progressive AD (AUC 0.946 ± 0.021 vs. 0.654 ± 0.057), and the NfL/p-tau ratio was the best biomarker in terms of sensitivity and specificity in this differential diagnosis (AUC 0.989 ± 0.007) (*see* Additional file 1: Figure S3).

## Discussion

We report the results of a comprehensive analysis of CSF NfL and other classical biomarkers in a large population of NDs, including, for the first time to our knowledge, almost all subtypes of prion disease and several cases of atypical/rapidly progressive AD, DLB, and FTLD. By confirming that all NDs are associated with a significant increase in CSF NfL levels compared with control subjects, our results support the contention that NfL represents a bona fide biomarker of neurodegeneration [22–29]. Specifically, sCJD and gCJD showed the highest values among NDs, as previously described [21, 22]. Moreover, by showing that NfL concentration in CSF is highly variable among prion disease subtypes and only partially correlates with t-tau levels, our data add to the knowledge of the mechanisms contributing to NfL elevation in CSF.

It is currently debated whether NfL elevation in CSF primarily reflects the degree of axonal (white matter) or synaptic (gray matter) pathology [27, 42, 43]. A correlation between NfL levels and white matter damage has been found in AD and vascular dementia [42]. Furthermore, on one hand, higher NfL levels in FTLD than in AD and DLB have been related to the prominent pathology in the frontal and temporal lobes that are rich in large-caliber axons in the former and the more severe involvement of subcortical regions in FTLD [26]. On the other hand, because neurofilament proteins are also integral components of synapses [43], NfL elevation may also reflect a synaptic origin of the disease process. In the present study, we have shown that sCJD subtypes VV2 and MV2K are characterized by significantly higher CSF NfL levels than the MM(V)1 and MM2C groups, as well as that a significant correlation between CSF NfL and t-tau levels is seen only in MM(V)1 and to a lesser extent in VV2 cases. Most significantly, whereas subjects with sCJD and MM(V)1 show significantly higher concentrations of CSF t-tau than MV2K patients, as previously reported [14, 15], the opposite is true for CSF NfL levels. Taken together, these results strongly suggest that NfL and t-tau reflect distinct pathophysiological mechanisms of neurodegeneration. We and others have previously shown that both VV2 and MV2K, compared with MM(V)1, are characterized by a more widespread, and on average more severe, subcortical pathology involving the hippocampus, amygdala, hypothalamus, and brainstem in addition to the striatum and medial thalamus [3, 31, 44]. Furthermore, the amount of PrP^Sc^ accumulation and microglial activation in subcortical white matter, including the cerebellum, is higher in VV2 and MV2K than in MM(V)1 [1].

**Fig. 2:**
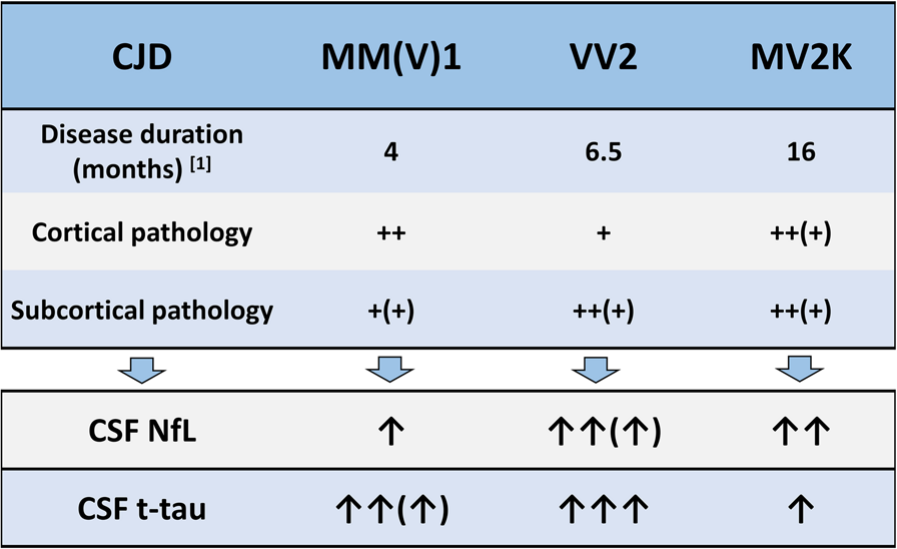
Heterogeneity of cerebrospinal fluid (CSF) neurofilament light chain protein (NfL) and total tau (t-tau) levels in Creutzfeldt-Jakob disease (CJD) MM1, VV2, and MV2K. Among CJD subtypes, CSF NfL and t-tau levels reflect both the rate of clinical progression and the relative extent of subcortical (deep nuclei, brainstem, and cerebellum) and cortical pathology

Thus, it seems that, at variance with t-tau, the concentration of CSF NfL is significantly influenced by the degree of subcortical axonal pathology, whereas both markers would reflect the extent of neuronal (i.e., gray matter) degeneration in a given time period. Accordingly, the latter mechanism will predominate in sCJD MM(V)1, explaining the good correlation between t-tau and NfL levels, whereas the former will be prominent in MV2K, the subtype with the slowest progressive course, which would fit with the significant increase in NfL levels despite the relatively low t-tau level. Finally, both the rapid course and extensive subcortical pathology would explain why sCJD VV2 is the neurodegenerative disorder associated with the highest NfL CSF levels (Fig. 2).

In our cohort, we also found that subjects with atypical/rapidly progressive FTLD demonstrate higher levels of NfL than typical cases. This is in line with previous studies showing a positive correlation between NfL values and disease severity in FTLD [24–27]; therefore, higher levels of NfL may predict a more rapid evolution of the disease in FTLD cases, even in the absence of comorbid white matter pathology (e.g., vascular). Interestingly, a rapidly progressive variant of FTLD characterized by a pure, widespread TDP-43 neuropathology has recently been described [45]. Future studies including neuropathologically verified atypical/rapidly progressive FTLD cases, unavailable in our series, are needed to determine whether patients affected by this variant indeed have increased NfL levels in CSF.

At variance with FTLD, we did not find any difference in NfL levels between typical and atypical/rapidly progressive variants of DLB and AD. Similarly, Llorens et al. detected comparable levels of CSF α-synuclein, a synaptic biomarker, in subjects with typical and rapidly progressive AD [46]. To date, no specific pathology, in either the gray or white matter, that could explain the more rapid course has been detected between typical and rapidly progressive DLB cases [6], and this extends to our present series of neuropathologically verified cases. In contrast, rapidly progressive AD has recently been linked to the presence of distinct Aβ structural conformers and to a different protein composition of amyloid plaques [47, 48]. Taken together, these data indicate that the pathophysiological mechanisms leading to a more rapid course in AD involve mainly the extracellular space of the cortical gray matter and are relatively independent from those affecting NfL and α-synuclein CSF levels [28, 29, 46].

Regarding the diagnostic value of CSF NfL, either alone or in combination with other biomarkers, we found that NfL equates t-tau in the overall discrimination of prion diseases from other NDs. Furthermore, NfL alone showed higher accuracy than t-tau in the distinction between atypical forms, although the t-tau/p-tau ratio demonstrated the best performance in this respect. Most significantly, we found that high levels of NfL were associated with virtually all sCJD subtypes, including those linked to PrP^Sc^ type 2 (MV2K, MM2C, and MM2T), which more often present with a negative 14-3-3 and/or RT-QuIC assay and/or low t-tau levels [14]. Therefore, in the diagnostic assessment of these subtypes, NfL may represent a useful, rapid test to complement the RT-QUIC prion assay. This is especially relevant in cases with negative cerebral MRI results, which are not so unusual in clinical practice. Finally, NfL and the NfL/p-tau ratio appeared to be superior to t-tau and the t-tau/p-tau ratio, respectively, in the distinction between prion diseases and AD overall, as well as between atypical/rapidly progressive AD and atypical prion disease, which further validates the diagnostic role of the NfL assay in NDs.

Considering CSF biomarker-based diagnosis of AD, CSF NfL did not significantly add to the value of t-tau, p-tau, and Aβ_42_, as previously described [49]. In contrast, we demonstrated good accuracy of NfL in combination with other biomarkers (NfL × Aβ_42_/p-tau) in the discrimination of FTLD cases from other typical dementias, such as AD and DLB.

We are aware that one potential limitation of our study is the relatively low number of neuropathologically verified cases of atypical/rapidly progressive NDs. Nevertheless, in all atypical/rapidly progressive cases, the final clinical diagnosis was formulated at follow-up after at least 2 years of further clinical observation. Moreover, our definition of atypical/rapidly progressive ND, though representing very well the real-world clinical scenario, could have introduced a source of bias because of its polymorphic nature (i.e., clinical, biochemical, and EEG criteria). In addition, the fact that CSF NfL levels may correlate with age [50] is unlikely to be of any relevance because all of our patients groups had comparable mean ages at the time of LP. Finally, our study was focused on NDs and did not take into account the diagnostic issues related to the rapidly progressive dementias secondary to vascular or inflammatory pathologies that are notoriously linked to increased NfL levels. Thus, the diagnostic significance of CSF NfL can be applied only to clinical situations in which neuroimaging and laboratory findings have consistently excluded such pathologies.

## Conclusions

Our data further validate CSF NfL as a neurodegenerative biomarker and provide further evidence for its distinctive diagnostic role and biological significance in a significant cohort of atypical/rapidly progressive NDs. Taken together, our data support the use of NfL as a fast screening marker for the differential diagnosis of rapidly progressive NDs, especially in the presence of atypical clinical and laboratory features.

## Additional file

Additional file 1: Table S1. Diagnostic value of CSF biomarkers in the comparison between subjects with ND and control subjects. **Table S2.** Diagnostic value of CSF biomarkers in the differential diagnosis of prion disease, AD, and FTLD. **Figure S1.** ROC analysis of CSF biomarkers in the comparison between prion disease and AD. **Figure S2.** ROC analysis of CSF biomarkers in the comparison between atypical prion disease and other atypical/rapidly progressive NDs. **Figure S3.** ROC analysis of CSF biomarkers in the comparison between atypical prion disease and atypical/rapidly progressive AD. (DOCX 1967 kb)

## Abbreviations

a/rp: Atypical/rapidly progressive
AD: Alzheimer’s disease
Aβ_42_: β-Amyloid 42
bvFTD: Behavioral variant of frontotemporal dementia
CBS: Corticobasal syndrome
CJD: Creutzfeldt-Jakob disease
CSF: Cerebrospinal fluid
CT: Computed tomography
DLB: Dementia with Lewy bodies
DW: Diffusion weighted
EEG: Electroencephalographic
ELISA: Enzyme-linked immunosorbent assay
FTD: Frontotemporal dementia
FTLD: Frontotemporal lobar degeneration
gCJD: Genetic Creutzfeldt-Jakob disease
GSS: Gerstmann-Sträussler-Scheinker syndrome
LP: Lumbar puncture
M: Methionine
MM2C: MM2 cortical
MM2T: MM2 thalamic
MRI: Magnetic resonance imaging
MV2K: MV2 kuru type
ND: Neurodegenerative dementia
NfL: Neurofilament light chain protein
PPA: Primary progressive aphasia
PrP^Sc^: Disease-associated prion protein
PSP: Progressive supranuclear palsy
PSWC: Periodic sharp wave complexes
p-tau: Phosphorylated tau
RT-QuIC: Real-time quaking-induced conversion
sCJD: Sporadic Creutzfeldt-Jakob disease
t-tau: Total tau
V: Valine
VPSPr: Variably protease-sensitive prionopathy
