# Supplementary material for: The CSF neurofilament light signature in rapidly progressive neurodegenerative dementias
Source: Alzheimers Res Ther. 2018 Jan 11;10:3. doi: 10.1186/s13195-017-0331-1 (PMC5784714; doi:10.1186/s13195-017-0331-1)
Supplement: Additional file 1: Table S1. — Diagnostic value of CSF biomarkers in the comparison between subjects with ND and control subjects. Table S2. Diagnostic value of CSF biomarkers in the differential diagnosis of prion disease, AD, and FTLD. Figure S1. ROC analysis of CSF biomarkers in the comparison between prion disease and AD. Figure S2. ROC analysis of CSF biomarkers in the comparison between atypical prion disease and other atypical/rapidly progressive NDs. Figure S3. ROC analysis of CSF biomarkers in the comparison between atypical prion disease and atypical/rapidly progressive AD. (DOCX 1967 kb) [file 13195_2017_331_MOESM1_ESM.docx]

**ADDITIONAL FILE**

**Table S1. Diagnostic value of CSF biomarkers in the comparison between NDs and controls.**

|  | **Prion disease vs. controls** | | | | | |  | **AD vs. controls** | | | | | |
| --- | --- | --- | --- | --- | --- | --- | --- | --- | --- | --- | --- | --- | --- |
|  | **AUC** |  | **cut-off** | **sens (%)** | **spec (%)** |  | | | **AUC** |  | **cut-off** | **sens (%)** | **spec (%)** |
| NfL | 1.000±0.001 | > | 2109 pg/ml | 99.2 | 100 |  | | | 0.849±0.046 | > | 1565 pg/ml | 76.7 | 78.9 |
| t-tau | 0.991±0.008 | > | 385 pg/ml | 98.5 | 100 |  | | | 0.993±0.006 | > | 314 pg/ml | 97.3 | 94.6 |
| t-tau/p-tau | 0.994±0.006 | > | 8.1 | 99.2 | 100 |  | | | 0.908±0.031 | > | 6.3 | 83.6 | 84.2 |
| NfL/p-tau | 0.984±0.009 | > | 75.7 | 94.6 | 94.7 |  | | | 0.724±0.062 | < | 25.9 | 68.4 | 65.8 |
|  |  |  |  |  |  |  | | |  |  |  |  |  |
|  | **DLB vs. controls** | | | | |  | | | **FTLD vs. controls** | | | | |
|  | **AUC** |  | **cut-off** | **sens (%)** | **spec (%)** |  | | | **AUC** |  | **cut-off** | **sens (%)** | **spec (%)** |
| NfL | 0.800±0.061 | > | 1592 pg/ml | 73.5 | 78.9 |  | | | 0.927±0.031 | > | 1792 pg/ml | 84.1 | 84.2 |
| t-tau | 0.680±0.073 | > | 235 pg/ml | 67.6 | 73.7 |  | | | 0.681±0.069 | > | 199 pg/ml | 63.6 | 57.9 |
| t-tau/p-tau | 0.640±0.075 | > | 5.7 | 55.9 | 63.2 |  | | | 0.691±0.066 | > | 5.6 | 61.4 | 57.9 |
| NfL/p-tau | 0.686±0.074 | > | 36.4 | 70.6 | 68.4 |  | | | 0.844±0.049 | > | 46.5 | 75.0 | 78.9 |

*AD* Alzheimer’s disease, *AUC* area under the curve, *DLB* dementia with Lewy bodies, *FTLD* frontotemporal lobar degeneration*, NfL* neurofilament light protein, *p-tau* phosphorylated tau protein, *sens* sensitivity, *spec* specificity, *t-tau* total tau protein

**Table S2. Diagnostic value of CSF biomarkers in the differential diagnosis of prion disease, AD and FTLD.**

|  | **Prion disease vs. DLB** | | | | | |  | **Prion disease vs. FTLD** | | | | | |
| --- | --- | --- | --- | --- | --- | --- | --- | --- | --- | --- | --- | --- | --- |
|  | **AUC** |  | **cut-off** | **sens (%)** | **spec (%)** |  | | | **AUC** |  | **cut-off** | **sens (%)** | **spec (%)** |
| NfL | 0.880±0.047 | > | 4069 pg/ml | 95.4 | 79.4 |  | | | 0.870±0.032 | > | 6237 pg/ml | 83.8 | 75.0 |
| t-tau | 0.963±0.015 | > | 1039 pg/ml | 89.2 | 88.2 |  | | | 0.982±0.009 | > | 741 pg/ml | 96.2 | 95.5 |
| t-tau/p-tau | 0.970±0.019 | > | 10.7 | 97.7 | 91.2 |  | | | 0.983±0.010 | > | 11.3 | 97.7 | 95.5 |
| NfL/p-tau | 0.863±0.049 | > | 104.3 | 92.3 | 79.4 |  | | | 0.786±0.044 | > | 155.7 | 75.4 | 70.5 |
|  |  |  |  |  |  |  | | |  |  |  |  |  |
|  | **AD vs. other NDs (DLB + FTLD)** | | | | |  | | |  | | | | |
|  | **AUC** |  | **cut-off** | **sens (%)** | **spec (%)** |  | | |  |  |  |  |  |
| NfL | 0.623±0.046 | < | 2160 pg/ml | 64.9 | 53.4 |  | | |  |  |  |  |  |
| t-tau | 0.879±0.029 | > | 465 pg/ml | 84.9 | 84.4 |  | | |  |  |  |  |  |
| p-tau | 0.919±0.023 | > | 65 pg/ml | 87.7 | 84.4 |  | | |  |  |  |  |  |
| Aβ42 | 0.775±0.039 | < | 441 pg/ml | 70.1 | 72.6 |  | | |  |  |  |  |  |
|  |  | | | | | |  |  | | | | | |
|  | **FTLD vs. other NDs (AD + DLB)** | | | | | |  | **FTLD vs. AD** | | | | | |
|  | **AUC** |  | **cut-off** | **sens (%)** | **spec (%)** |  | | | **AUC** |  | **cut-off** | **sens (%)** | **spec (%)** |
| NfL | 0.698±0.050 | > | 2525 pg/ml | 69.8 | 61.7 |  | | | 0.716±0.053 | > | 2656 pg/ml | 62.8 | 64.4 |
| t-tau | 0.792±0.039 | < | 384 pg/ml | 74.8 | 74.4 |  | | | 0.910±0.030 | < | 485 pg/ml | 83.6 | 86.0 |
| p-tau | 0.824±0.034 | < | 52 pg/ml | 77.6 | 72.1 |  | | | 0.939±0.022 | < | 66 pg/ml | 86.3 | 86.0 |
| Aβ42 | 0.796±0.042 | > | 502 pg/ml | 79.1 | 74.8 |  | | | 0.860±0.040 | > | 495 pg/ml | 79.1 | 84.9 |
| NfL× Aβ42/p-tau | 0.900±0.025 | > | 25405 | 83.7 | 81.3 |  | | | 0.975±0.011 | > | 18638 | 90.7 | 94.5 |

*Aβ42* beta-amyloid 42, *AD* Alzheimer’s disease, *AUC* area under the curve, *DLB* dementia with Lewy bodies, *FTLD* frontotemporal lobar degeneration*, NfL* neurofilament light protein, *p-tau* phosphorylated tau protein, *sens* sensitivity, *spec* specificity, *t-tau* total tau protein

**Figure S1. ROC analysis for CSF biomarkers in the comparison between prion disease and AD.**


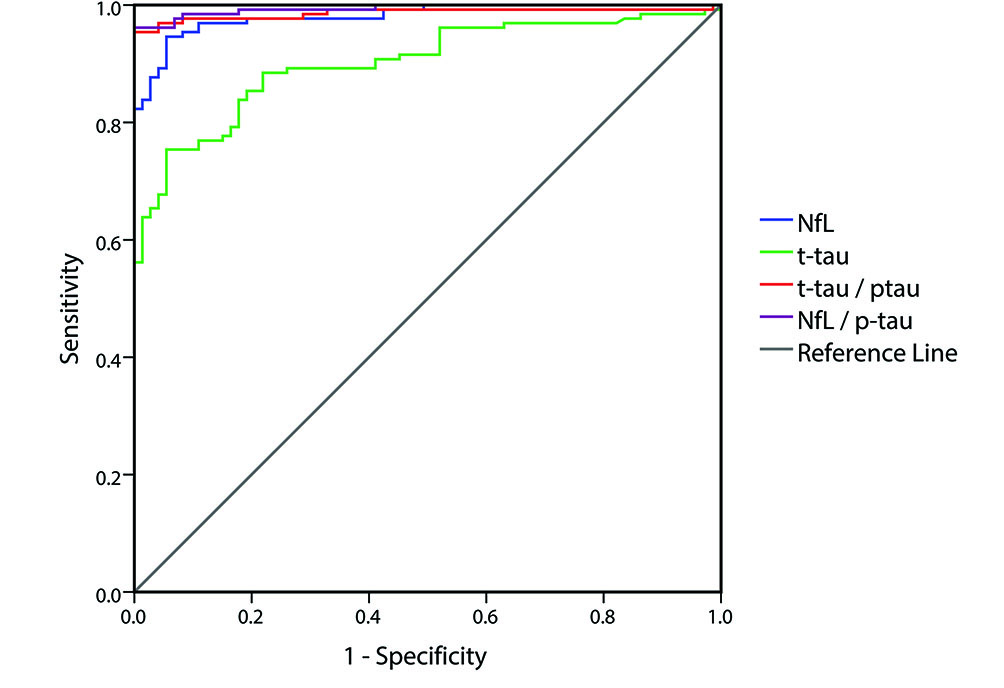


**Figure S2. ROC analysis for CSF biomarkers in the comparison between atypical prion disease and other atypical/rapidly progressive NDs.**


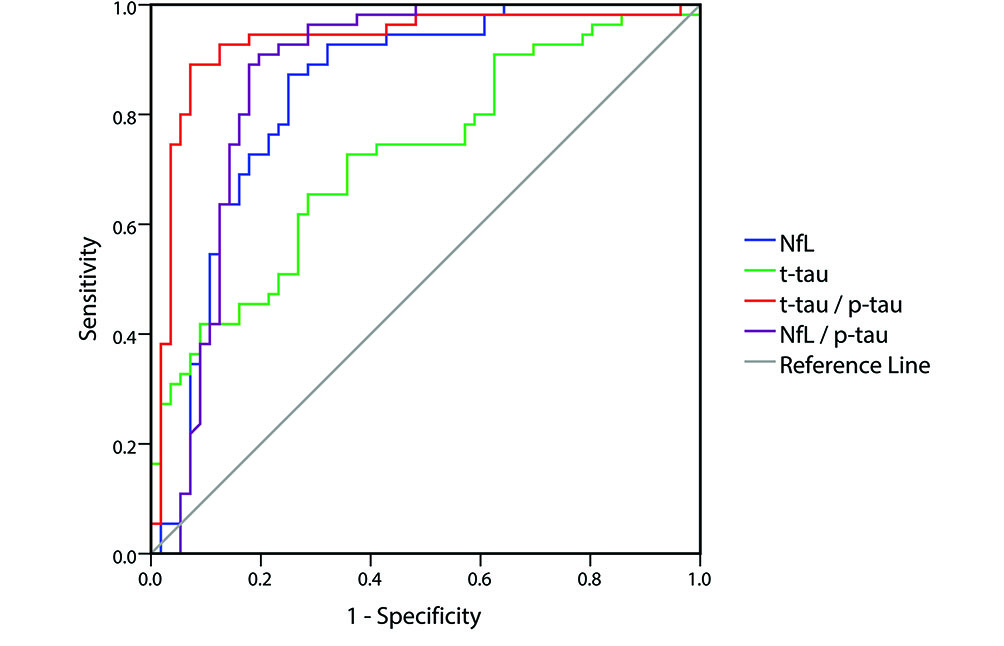


**Figure S3. ROC analysis for CSF biomarkers in the comparison between atypical prion disease and atypical/rapidly progressive AD.**

**
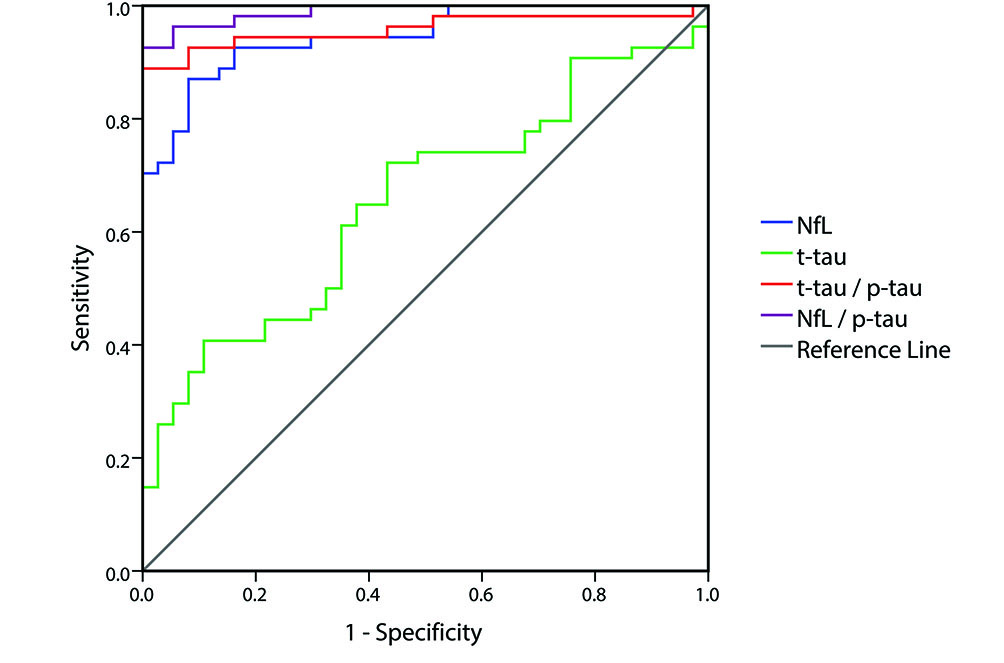
**
